# Maximization of non-idle enzymes improves the coverage of the estimated maximal *in vivo* enzyme catalytic rates in *Escherichia coli*

**DOI:** 10.1093/bioinformatics/btab575

**Published:** 2021-08-06

**Authors:** Rudan Xu, Zahra Razaghi-Moghadam, Zoran Nikoloski

**Affiliations:** Bioinformatics, Institute of Biochemistry and Biology, University of Potsdam, 14476 Potsdam, Germany; Bioinformatics, Institute of Biochemistry and Biology, University of Potsdam, 14476 Potsdam, Germany; Systems Biology and Mathematical Modelling Group, Max Planck Institute of Molecular Plant Physiology, 14476 Potsdam, Germany; Bioinformatics, Institute of Biochemistry and Biology, University of Potsdam, 14476 Potsdam, Germany; Systems Biology and Mathematical Modelling Group, Max Planck Institute of Molecular Plant Physiology, 14476 Potsdam, Germany

## Abstract

**Motivation:**

Constraint-based modeling approaches allow the estimation of maximal *in vivo* enzyme catalytic rates that can serve as proxies for enzyme turnover numbers. Yet, genome-scale flux profiling remains a challenge in deploying these approaches to catalogue proxies for enzyme catalytic rates across organisms.

**Results:**

Here, we formulate a constraint-based approach, termed NIDLE-flux, to estimate fluxes at a genome-scale level by using the principle of efficient usage of expressed enzymes. Using proteomics data from *Escherichia coli*, we show that the fluxes estimated by NIDLE-flux and the existing approaches are in excellent qualitative agreement (Pearson correlation > 0.9). We also find that the maximal *in vivo* catalytic rates estimated by NIDLE-flux exhibits a Pearson correlation of 0.74 with *in vitro* enzyme turnover numbers. However, NIDLE-flux results in a 1.4-fold increase in the size of the estimated maximal *in vivo* catalytic rates in comparison to the contenders. Integration of the maximum *in vivo* catalytic rates with publically available proteomics and metabolomics data provide a better match to fluxes estimated by NIDLE-flux. Therefore, NIDLE-flux facilitates more effective usage of proteomics data to estimate proxies for kcatomes.

**Availability and implementation:**

https://github.com/Rudan-X/NIDLE-flux-code.

**Supplementary information:**

Supplementary data are available at *Bioinformatics* online.

## 1 Introduction

The turnover number, $k_{\mathrm{cat}}$, is a fundamental characteristic of enzymes. Documenting the kcatome, i.e. the collection of turnover numbers of all proteins with enzymatic function (Nilsson *et al.*, 2017), has led to improved accuracy of predicted metabolic phenotypes, can be employed to design feasible metabolic engineering strategies (Adadi *et al.*, 2012; Beg *et al.*, 2007; Sánchez *et al.*, 2017), and will propel the creation of kinetic metabolic models at a larger scale (Khodayari and Maranas, 2016). Integration of the kcatome in constraint-based modeling approaches have also lead to the generation of resource allocation models in bacteria (Bulović, *et al.*, 2019) and models that incorporate macromolecular expression (ME) for *Escherichia coli* (Fang, *et al.*, 2020), which have led to predictions about enzyme allocations in the studied organisms under different conditions. However, measurements of the kcatome based on *in vitro* characterization with classical techniques are limited due to impossibility to purify specific enzymes, lack of availability of substrates and knowledge of required cofactors. In addition, the relevance of the *in vitro* obtained parameters for studies of *in vivo* phenotypes remains debatable (van Eunen and Bakker, 2014; Labhsetwar *et al.*, 2017).

Recent advances in computational approaches to integrate quantitative proteomics data in large-scale models of metabolism have facilitated the estimation of *in vivo* proxies for the kcatomes of *E.coli and Arabidopsis thaliana* (Davidi *et al.*, 2016; Heckmann, *et al.*, 2020; Küken *et al.*, 2020). One way to obtain *in vivo* proxies of the kcatome is to rely on the relation between metabolic flux, $v\left(C\right),\;$and enzyme abundance, $E\left(C\right)$, in a given condition C. More specifically, $v\left(C\right)=k_{\mathrm{cat}}\eta \left(C\right)E(C)$, with $\eta \left(C\right)$ denoting the effect of metabolite concentrations and other parameters (e.g. $K_{M}$ in Michaelis–Menten kinetics). As a result, $k_{\mathrm{app}}(C)=k_{\mathrm{cat}}\eta \left(C\right)$, can be obtained by the ratio $v\left(C\right)/E(C)$. When data from multiple conditions are available, Davidi *et al.* (2016) defined the maximal *in vivo* catalytic rate $k_{\mathrm{vivo}}^{\max}$ as the maximum of the $k_{\mathrm{app}}(C)$ over the considered conditions. The same approach can be used when data from knock-out strains (undergoing subsequent adaptive laboratory evolution) are used instead, in which case $k_{\mathrm{vivo}}^{\max}$ is the maximum over the considered strains (Heckmann *et al.*, 2020). Due to enzyme saturation, the value of $\eta \left(C\right)$ is bounded from above by 1 (i.e. $0\leq \eta \left(C\right)\leq 1$), and thus $k_{\mathrm{vivo}}^{\max}$ represents a lower bound for $k_{\mathrm{cat}}$. This approach can be used to estimate $k_{\mathrm{vivo}}^{\max}$ for homomeric enzymes and has also been employed to estimate the average $k_{\mathrm{vivo}}^{\max}$ of heteromeric enzymes and iso-enzymes (Davidi *et al.*, 2016).

Applications of these approaches have shown that $k_{\mathrm{vivo}}^{\max}$ generally concur with *in vitro* $k_{\mathrm{cat}}$ values (by using Pearson correlation of log-transformed values) (Davidi *et al.*, 2016), and that integration of the resulting $k_{\mathrm{vivo}}^{\max}$ in protein-constrained models leads to more precise predictions of enzyme abundances in comparison to in vitro $k_{\mathrm{cat}}$ values (Heckmann *et al.*, 2020). However, assuming that the proteomics data are obtained with the same profiling technology (thus minimizing technical artifacts), the accuracy of these approaches depends on the values of the estimated fluxes. In the calculation of $k_{\mathrm{vivo}}^{\max}$, Davidi *et al.* (2016) used flux estimates obtained by applying parsimonious FBA (pFBA) (Lewis *et al.*, 2010). This constraint-based approach predicts a flux distribution of minimum total flux compatible with constraints on growth, uptake rates and fluxes of specific reactions from ^13^C labeling studies. While fluxes from pFBA have been shown to result in precise estimates of $k_{\mathrm{vivo}}^{\max}$, due to the low variability of fluxes, it is still not clear to what extent these flux values reflect the actual reaction rates *in vivo*. To overcome this issue, Heckmann *et al.* (2020) relied on flux estimates obtained from integration of ^13^C labeling patterns in a metabolic model following ^13^C metabolic flux analysis (^13^C-MFA) (McCloskey *et al.*, 2018a,b,c,d). However, it is known that the estimation of fluxes with approaches from ^13^C-MFA strongly depends on the model used for fitting of the labeling patterns, arguing for usage of genome-scale models to reduce bias of flux estimates (Gopalakrishnan *et al.*, 2018).

Despite these differences, both approaches share a striking similarity: Davidi *et al.* (2016) found almost a third of the homomeric enzymes with non-zero abundance not to carry flux in any of the investigated conditions. These so-called *idle enzymes* are attributed as an artifact of the pFBA approach used for *in silico* flux estimation, and are not included in the estimation of $k_{\mathrm{vivo}}^{\max}$. Although Heckmann *et al.* (2020) rely on fluxes obtained from ^13^C-MFA, they also estimate $k_{\mathrm{vivo}}^{\max}$ only for those homomeric enzymes that are expressed above 50 pmol/gDW and for which the corresponding reactions are not blocked, determined by an arbitrary threshold. In addition, for both approaches it remains unclear if the corresponding idle enzymes are of substantial abundance.

Several experimental and theoretical studies have pointed out that protein production contributes predominantly to cellular costs (Dourado and Lercher, 2020; Hui *et al.*, 2015; Klumpp *et al.*, 2013; Molenaar *et al.*, 2009; Weiße *et al.*, 2015). Under the evolutionary favored assumption of effective usage of enzymes, as costly cellular resources, it is expected that expressed enzymes carry flux. However, such a constraint is not considered in the existing constraint-based approaches for flux estimation. Having accesses to proteomics data, this problem can be addressed by devising a computational approach for flux estimation that maximizes the number of expressed enzymes that carry flux while respecting the input-output constraints. In such a way, one can maximize the number of enzymes for which $k_{\mathrm{vivo}}^{\max}$ can be obtained, thus increasing the coverage of the proxy for the kcatome. While this approach may bias the estimates, due to consideration only of proteins with measured abundance, the bias is minimized due to the increasing coverage of the proteomics measurements with modern technologies (Messner *et al.*, 2019).

The idea of maximizing the number of expressed enzymes that carry flux has been used in the extraction of context-specific metabolic models (Robaina-Estévez and Nikoloski, 2014), particularly in the so-called iMAT-family (Agren *et al.*, 2014; Shlomi *et al.*, 2008) and MBA-family (Jerby *et al.*, 2010; Vlassis *et al.*, 2014; Wang *et al.*, 2012) of constraint-based approaches. While these approaches are suitable for identification of subset of reactions compatible with enzymes whose abundance exceeds a threshold, they are not designed to obtain flux estimates in the scenario from which enzyme abundance data are obtained. In addition, there is usually a large number of alternative solutions compatible with the constraints imposed by these approaches, as demonstrated on several comparative studies (Robaina-Estévez and Nikoloski, 2017), posing further challenges regarding the precision of flux estimates.

To overcome the aforementioned issues, here we propose a constraint-based approach, called maximization of non-idle enzymes, abbreviated as NIDLE-flux. It is based on the principle that cells have evolved towards effective usage of costly cellular resources, like enzymes. We use the fluxes obtained from NIDLE-flux with constraints from different conditions and strains to provide estimates of $k_{\mathrm{vivo}}^{\max}$ in *E.coli*. Through comprehensive comparative analyses with corresponding proteomics data, we demonstrate that the estimated $k_{\mathrm{vivo}}^{\max}$ values obtained from NIDLE-flux are in concordance with the *in vitro* $k_{\mathrm{cat}}$ values as well as $k_{\mathrm{vivo}}^{\max}$ estimated from the contenders. Our findings show that NIDLE-flux leads to a 1.4-fold increase in the coverage of the estimated proxies for the kcatome, without compromising the quality and precision of the estimates.

## 2 Materials and methods

### 2.1 Maximizing the number of reactions that carry flux compatible with protein abundance

In this section, we propose a two-step constraint-based approach that integrates quantitative proteomics data along with constraints on nutrient uptake and growth rate to estimate fluxes by maximizing the number of non-idle enzymes, termed NIDLE-flux, under specified growth conditions. In the first step, NIDLE-flux maximizes the number of reactions that support flux and are associated with active gene–protein-reaction (GPR) rules according to the set of enzymes expressed with non-zero abundance, denoted by$R_{a}$. The GPR rules are applied by considering the maximum abundance among isoenzymes and minimum among the units of a protein complex. In contrast to the aforementioned approaches for context-specific model extractions, we first split all reversible reactions into forward and backward reactions. Each irreversible reaction with an active GPR state (based on non-zero abundance after applying the GPR rule) is in turn associated an integer variable, $y_{j}$, while keeping track of the corresponding forward and backward reactions. We impose the constraint that only one of these reactions carries flux (Equation 5), corresponding to the net-flux, $v_{j}=v_{j,\mathrm{for}}-v_{j,\mathrm{back}}$, of the original reversible reaction, under the default lower and upper bounds, $v_{\min,j}$ and $v_{\max,j}$ respectively. Flux capacity constraints, including nutrient uptake, are given in Equation (2) (with $v_{\max,i}$ a default upper bound), while constraints on growth are given in Equation (3), with μ denoting the measured growth rate, and the small range employed to prevent infeasibilities in applications of the generic formulation of the approach. In the generation of our results, Equations (3) and (10), below, are implemented with $v_{\mathrm{biomass}}=\;\mu$. As a result, NIDLE-flux is formulated as a mixed-integer linear program (MILP):

$$z=\max\sum_{j\;\in R_{a}}y_{j}$$

 

(1) s.t. N·v=0

 

(2) $$0\leq v_{i}\leq v_{\max,i}$$

 

(3) $$0.95\cdot \mu \;\leq v_{\mathrm{biomass}}\leq 1.05\cdot \mu$$

 

(4) $$v_{\min,j\;}\cdot \left(1-y_{j}\right)\;+\;\epsilon \cdot y_{j}\leq v_{j}{\leq y}_{j}\cdot v_{\max,j}+\epsilon \cdot \left(1-y_{j}\right),\;j\;\in R_{a}$$

 

(5) $$y_{j,\mathrm{for}}+\;y_{j,\;\mathrm{back}}\leq 1,j\;\mathrm{is}\;a\;\mathrm{reversible}\;\mathrm{reaction}\;\mathrm{in}\;R_{a}$$

 

(6) $$y_{j}\in \left\{0,1\right\},\;j\;\in R_{a}$$

Importantly, by integrating the proteomics data in a genome-scale metabolic model, NIDLE-flux does not assume proportionality between flux and protein abundance. Moreover, as long as an enzyme has been reported as measured, and thereby over the detection limit of the measuring technology, it is used to set up the constraints of the MILP in Equations (1)–(6).

In the second step, to obtain flux estimates NIDLE-flux minimizes the total flux of active reactions, while fixing the optimum number of active reactions, z. This is commonly applied strategy to narrow down the solution space, and has the following MILP formulation:

$$q=\min\sum_{i}v_{i}$$

 

(7) s.t. N·v=0

 

(8) $$0\leq v_{i}\leq v_{\max,i}$$

 

(9) $$v_{\mathrm{other}\;\mathrm{carbon}\;\mathrm{sources}}=0$$

 

(10) $$0.95\cdot \mu \;\leq v_{\mathrm{biomass}}\leq 1.05\cdot \mu$$

 

(11) $$v_{\min,j\;}\cdot \left(1-y_{j}\right)\;+\;\epsilon \cdot y_{j}\leq v_{j}{\leq y}_{j}\cdot v_{\max,j}+\epsilon \cdot \left(1-y_{j}\right)\;j\;\in R_{a}$$

 

(12) $$y_{\mathrm{for},j}+\;y_{\mathrm{back},j}\leq 1\;j\;\in R_{a}$$

 

(13) $$\sum_{j}y_{j}=\;z.$$

The formulation of NIDLE-flux may favor the inclusion of reactions with inactive GPR state. To control for this possibility, we also designed a penalized version of the approach, whereby the objective z is given by

$$z=\max\sum_{j\;\in R_{a}}y_{j}-\sum_{j\;\in R_{i}}y_{j},$$

where $R_{i}$ denotes the set of reactions with inactive GPR rules. We refer to this version of NIDLE-flux as NIDLE-penalized.

In contrast to NIDLE-flux, which is formulated as a two-step MILP-based approach, parsimonious FBA (pFBA) aims at minimizing the total flux in the network in the specified growth condition (Lewis *et al.*, 2010). The GitHub implementation of NIDLE-flux and NIDLE-penalized, available at https://github.com/Rudan-X/NIDLE-flux-code, also includes an implementation of pFBA and exploration of the space of alternative solutions.

### 2.2 Flux variability analysis for NIDLE-flux and pFBA

To investigate the precision of the flux estimated by NIDLE-flux, flux variability analysis (FVA) was performed for every reaction that carries non-zero flux and is associated with known enzyme abundance. To conduct FVA, we determined the minimum and maximum values of flux for each active reaction, assuming that the number of active reactions and total flux are fixed to the solutions of the preceding two MILPs:

min/max ⁡vj j ∈Ra

 

(14) s.t. N·v=0

 

(15) $$0\leq v_{i}\leq v_{\max,i}$$

 

(16) $$v_{\mathrm{other}\;\mathrm{carbon}\;\mathrm{sources}}=0$$

 

(17) $$0.95\cdot \mu \;\leq v_{\mathrm{biomass}}\leq 1.05\cdot \mu$$

 

(18) $$v_{\min,j\;}\cdot \left(1-y_{j}\right)\;+\;\epsilon \cdot y_{j}\leq v_{j}{\leq y}_{j}\cdot v_{\max,j}+\epsilon \cdot \left(1-y_{j}\right)\;j\;\in R_{a}$$

 

(19) $$\sum_{j}y_{j}=\;z$$

 

(20) $$\sum_{i}v_{i}=\;q.$$

We note that all results are obtained by using an equality constraint in Equation (17), whereby the flux through the biomass reaction is fixed to the measured growth rate. This was also the case for Equations (24) and (28), below.

pFBA was also implemented to estimate the fluxes under different growth condition constrains (the same first five constraints as in MILP) with the objective of minimizing the total flux carried by all reactions in the network. Similar idea was applied to assess the variability each flux, under the additional constraint that the total flux equals to the minimum obtained from pFBA:

$$q^{'}=\min\sum v_{i}$$

 

(21) s.t. N·v=0

 

(22) $$0\leq v_{i}\leq v_{\max,i}$$

 

(23) $$v_{\mathrm{other}\;\mathrm{carbon}\;\mathrm{sources}}=0$$

 

(24) $$0.95\cdot \mu \;\leq v_{\mathrm{biomass}}\leq 1.05\cdot \mu .$$

In the FVA for pFBA, an additional constraint was imposed by fixing the total flux to the optimal solution of the pFBA as follows:

min/max ⁡vj j ∈Ra

 

(25) s.t. N·v=0

 

(26) $$0\leq v_{i}\leq v_{\max,i}$$

 

(27) $$v_{\mathrm{other}\;\mathrm{carbon}\;\mathrm{sources}}=0$$

 

(28) $$0.95\cdot \mu \;\leq v_{\mathrm{biomass}}\leq 1.05\cdot \mu$$

 

(29) $$\sum_{i}v_{i}=q^{'}.$$

### 2.3 Model and data-driven metabolic constraints

The genome-scale metabolic model of *E.coli* (iJO1366) was converted into an irreversible model, whereby reversible reactions are split into two irreversible reactions. For both MILP and pFBA approaches, constraints imposed to the model are based on proteomic studies of *E.coli* in 31 different conditions (Peebo *et al.*, 2015; Schmidt *et al.*, 2016; Valgepea *et al.*, 2013). The data specify the composition of the media and measured growth rate in each condition (see Supplementary Table S1).

### 2.4 $k_{\mathrm{vivo}}^{\max}$ estimation and idle enzymes based on proteomics data

Given data on protein abundance in condition C and a steady-state flux distribution, v(C), determined by a selected approach, we first estimate $k_{\mathrm{app}}\left(C\right)=v\left(C\right)/E(C)$ for a reaction catalyzed by this enzyme. The simplicity of calculation is due to the fact that only reactions catalyzed by a unique homomeric enzyme are considered. Reactions that carry flux in $v\left(C\right)\;$but do not have measured abundance in E(C) are not considered in the calculations. The maximum of $k_{\mathrm{app}}\left(C\right)$ over all conditions is defined as the maximal *in vivo* catalytic rate, denoted by $k_{\mathrm{vivo}}^{\max}$. For the comparison with the findings from the ^13^C-MFA study (Heckmann *et al.*, 2020), two biological replicates were provided, and the estimate of $k_{\mathrm{vivo}}^{\max}$ is given by the average of the respective $k_{\mathrm{vivo}}^{\max}$ values.

Here, we consider a reaction as active if it carries flux larger than $10^{-10}$ mmol (gDW h)^-^^1^ (see Supplementary Material for analysis of robustness for threshold $10^{-5}$ mmol (gDW h)^-^^1^).

## 3 Results

### 3.1 Comparative analysis of idle enzymes in NIDLE-flux, pFBA and ^13^C-MFA

We used NIDLE-flux with *E.coli’*s genome-scale metabolic network reconstruction iJO1366 (Orth *et al.*, 2011) under the constraints provided by experimental data (i.e. carbon source, growth rate and protein abundance) gathered from 31 conditions (Peebo *et al.*, 2015; Schmidt *et al.*, 2016; Valgepea *et al.*, 2013), employed in the study of Davidi *et al.* (2016). In addition, we applied NIDLE-flux to estimate steady-state flux distributions in 18 evolutionarily adapted knock-out strains by considering uptake, secretion, growth rates and protein abundances as constraints, as in the ^13^C-MFA analysis (McCloskey *et al.*, 2018a,b,c,d) (Supplementary Table S1).

Given the set of measured enzyme abundance across 31 conditions, the abundance of 903 enzymes was measured in at least one of the conditions. From these, 479 enzymes could be mapped to 1030 reactions with active GPR rules, each containing a single gene (Supplementary Table S1). Together with the rest of expressed enzymes, we found that an average of 1737 reactions were associated with active GPR state and were used in the estimation of fluxes. For the 31 growth conditions, NIDLE-flux activated an average of 603 reactions, while pFBA could do so for ∼29% fewer (i.e. 427) reactions.

Moreover, in the ^13^C-MFA study, we used two biological replicates for each endpoint strain, and found 584 enzymes to be expressed in at least one of the strains and one of the replicates; all of them could be mapped uniquely to a total of 1263 reactions. By applying the same rules to the reactions associated with these enzymes, an average of 1207 and 1212 reactions, in the respective replicates, were considered for flux estimation. There were on average 443 active reactions estimated by pFBA in the scenario with the 18 strains; in contrast, NIDLE-flux activated on average 665 and 673 reactions in the two replicates of the 18 strains, respectively. Interestingly, ^13^C-MFA estimated fluxes for an average of 360 reactions (∼46% fewer reactions than NIDLE-flux) over the 18 strains, based on the rules of considering a reaction non-blocked in Heckmann *et al.* (Heckmann *et al.*, 2020) (see Supplementary Table S2). These findings demonstrated that NIDLE-flux activates more reactions in comparison to the two contending approaches.

**Fig. 1.**
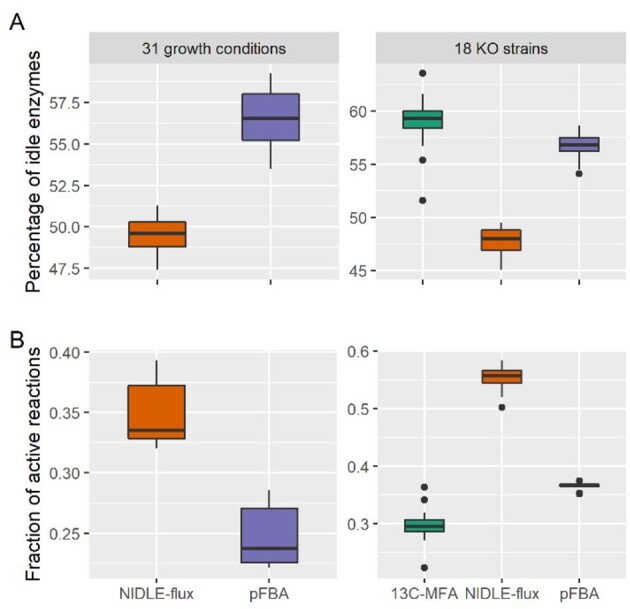
Comparison of contending approaches based on idle enzymes. Comparison of NIDLE-flux with pFBA on data from 31 different growth conditions and with ^13^C-MFA on data from 18 strains with respect to: (**A**) percentage of idle enzymes; NIDLE flux results in smaller percentage in comparison to the contending approaches (*P*-values < 10^*−*^^5^). (**B**) the fraction of the number of reactions carrying non-zero flux and the number of reactions with active GPR state, i.e. associated with expressed enzymes; NIDLE-flux activates more reactions per enzyme with measured abundance in comparison to the two contending approaches (*P*-values < 10^*−*^^6^)

Next, we asked how many idle enzymes were estimated by the employed approaches, i.e. how many enzymes are expressed, but their catalyzed reactions with active GPR state do not carry flux (see Section 2). We found that, on average, ∼57% and 59% of enzymes in pFBA and ^13^C-MFA were idle over 31 conditions and 18 strains (with two replicates), respectively (Fig. 1A, Supplementary Table S3). In contrast, on average ∼50% and 48% of enzymes were predicted as idle over the 31 conditions and 18 strains, respectively, when fluxes are estimated by NIDLE-flux. Therefore, NIDLE-flux results in a smaller number of idle enzymes compared with pFBA and ^13^C-MFA.

We also observed that the ratios of the number of active reactions to the number of reactions associated with expressed enzymes in pFBA and ^13^C-MFA were smaller than the ratio for NIDLE-flux (*P*-values = $1.4\times 10^{-11}$ and $3.2\times 10^{-7}$, respectively, see Fig. 1B). This observation indicated that, in ensuring that the physiological constraints are met, NIDLE-flux activates a larger number of reactions with measured abundance in comparison to pFBA and ^13^C-MFA, as expected from the formulation of the optimization problem. We also found that NIDLE-flux results in a smaller relative abundance of idle enzymes calculated from the data obtained from the experiments with 18 strains, in comparison to the contending approaches (Supplementary Fig. S1 and Supplementary Table S3).

Moreover, the findings regarding the percentage of idle enzymes and fraction of active reactions associated with enzymes of measured abundance were robust with respect to the usage of a larger threshold for considering a reaction non-blocked (Section 2, Supplementary Figs S2 and S3). These results indicated that NIDLE-flux indeed results in flux distributions that provides a more efficient usage of expressed enzymes.

We also compared the difference between the medians of the fluxes that were considered active in NIDLE-flux, but for which no protein abundance was measured. We found that on average for ∼437 active reactions with GPR rules over the 31 conditions there were protein abundance data available, while this was not the case for on average ∼110 active reactions with GPR rules. Interestingly, and most importantly, in all 31 conditions, the median flux carried by the active reactions without protein abundance data was at least three orders of magnitude smaller than the median flux carried by active reactions with protein abundance. In addition, we found that on average for ∼408 active reactions with GPR rules over the 18 knock-out strains there were protein abundance data available, while this was not the case for on average ∼201 active reactions with GPR rules. In contrast to the scenario with the 31 conditions, here we do not identify a statistically significant difference in the median fluxes carried by the active reactions with and without protein abundance data. The reason for this finding is likely due to the larger number of activated reactions, since we do not consider any thresholds on protein abundance for reaction activation.

### 3.2 Comparative analysis of fluxes estimated from NIDLE-flux and contending approaches

**Fig. 2.**
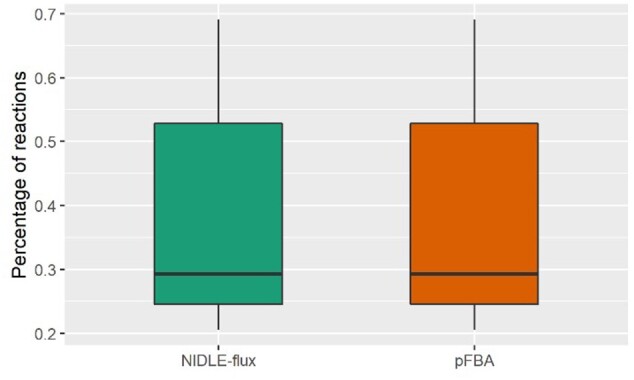
Agreement of flux estimates of NIDLE-flux and pFBA with ^13^C-MFA. The comparison is based on reactions with flux estimates by all three approaches, namely, NIDLE-flux, pFBA and ^13^C-MFA. Shown are the percentages of reactions in pFBA and NIDLE-flux whose fluxes fall in the confidence intervals obtained from ^13^C-MFA

Assuming that technical artifacts in measuring proteomics data are well-controlled, the main differences in the estimated maximal *in vivo* enzyme catalytic rates stems from the flux estimates. Therefore, having predictions about fluxes that obey the imposed input-output constraints, we next investigated the concordance of the flux estimates from the three compared approaches. First, we determined the percentage of fluxes estimated from pFBA and NIDLE-flux over the 18 strains whose value is in the range provided by the confidence interval from ^13^C-MFA (see Supplementary Table S2). The comparison that includes reactions for which there are flux estimates by all three approaches demonstrated that only a small percentage of, on average, both 37% in pFBA and NIDLE-flux quantitatively agree with the estimates from ^13^C-MFA. This was not surprising, given that pFBA and NIDLE-flux estimates were obtained from a different model than those of ^13^C-MFA (Fig. 2 and Supplementary Table S2).

Since the comparison of the estimated maximal *in vivo* catalytic rates was carried out in a qualitative manner, by considering the correlation of log-transformed values (due to the difference in the order of magnitude), we next evaluated the concordance between the flux estimates of pFBA and NIDLE-flux with those of ^13^C-MFA. Due to the observations above, about the differences between the sets of active reactions, for fair comparison we next focused on those reactions that were estimated as active by all three approaches. We found that the average correlation of flux predictions (on a log-scale) from NIDLE-flux and pFBA with flux estimates from ^13^C-MFA was high (minimum > 0.9667) in both comparisons (Supplementary Fig. S4 and Supplementary Table S2). Interestingly, we could identify steady-state flux distributions that obey the constraints provided by the confidence intervals from the ^13^C-MFA for the reactions considered active by all three approaches. The active reactions estimated by pFBA and ^13^C-MFA have a high minimum and average correlation of 0.97 and 0.99. The reactions shared by pFBA and ^13^C-MFA coincided with those shared by all three approaches, thus explaining the high correlation observed. In contrast, we identified a minimum and average Pearson correlation (log-scale) of 0.31 and 0.45 between the active reactions shared by NIDLE-flux and ^13^C-MFA (Supplementary Table S2). The reason for this discrepancy stems from the fact that there is no steady-state flux distribution that obeys the constraints from the confidence intervals from ^13^C-MFA when considering the active reactions in common with NIDLE-flux. Altogether, these results indicated that the flux estimates for NIDLE-flux could be employed in other analyses, including the estimation of proxies for the kcatome.

In addition, we determined the percentage of the active reactions from NIDLE-flux that are also active in each of the two contending approaches (see Supplementary Fig. S5); interestingly, more than 98% of the active reactions in pFBA were also active in NIDLE-flux. However, only 30% of the reactions deemed active in ^13^C-MFA were also active in NIDLE-flux. The reason for the latter finding is that the fluxes in ^13^C-MFA are determined from a different model, which can have large effects on the flux estimations (Gopalakrishnan *et al.*, 2018). The reactions that were activated only by NIDLE-flux, but not by pFBA in at least one of the conditions were enriched in reactions that participate in alternate carbon metabolism, cofactor and prostetic group biosynthesis, nucleotide salvage pathway, oxidative phosphorylation, lipopolysaccharide biosynthesis, murein recycling, glycolysis/gluconeogenesis and various transport reactions (see Supplementary Table S4). The reactions that were activated only by NIDLE-flux, but not found active by ^13^C-MFA were enriched for reactions that participate in different amino acid biosynthesis pathways, glycolysis/gluconeogenesis, pentose phosphate pathway, murein recycling and various transport processes (see Supplementary Table S4).

### 3.3 Comparative analysis of the estimates of the maximal *in vivo* enzyme catalytic rates

In the following, we compared the number of non-zero estimates of $k_{\mathrm{app}}(C)$ with available proteome data across the growth conditions and strains, denoted by C (see Section 2). For a fair comparison with the results from the studies of Davidi *et al.* (2016) and Heckmann *et al.* (2020), we focused on reactions catalyzed only by homomeric enzymes. More specifically, we asked if NIDLE-flux results in a larger number of estimated $k_{\mathrm{vivo}}^{\max}$ values. In the ^13^C-MFA study, two biological replicates were selected for each of the knock-out strains yielding two abundance profiles. Therefore, the $k_{\mathrm{vivo}}^{\max}$ value was the average between the two sets. In both sets of experiments, the number of $k_{\mathrm{app}}(C)$ estimates were consistently larger than those from pFBA and ^13^C-MFA (Tables 1 and Supplementary Table S4). The number of estimates obtained by pFBA did not change over the considered proteomics datasets, while NIDLE-flux considered the available variation in proteins with measured abundance. As a result, the standard deviation of $k_{\mathrm{app}}(C)$ estimates was larger for NIDLE-flux due to the variation and the larger number of proteins whose abundance was considered. However, this also led to a larger number of $k_{\mathrm{vivo}}^{\max}$ estimated by NIDLE-flux is comparison to pFBA when data over different growth conditions were considered. These findings demonstrated that NIDLE-flux can be employed in the estimation of proxies for catalytic rates for more reactions (Table 1). Interestingly, these results remained similar with the different threshold value for considering a reaction non-blocked (see Supplementary Table S5).

**Table 1.** Statistics for the number of estimated $k_{\mathrm{app}}(C)$ and $k_{\mathrm{vivo}}^{\max}$

**Cardinality of** $k_{\mathrm{app}}$								
	31 growth conditions		KO strains ALE					
	pFBA	NIDLE-flux	^13^C-MFA(1)	^13^C-MFA(2)	pFBA(1)	pFBA(2)	NIDLE-flux (1)	NIDLE-flux(2)
Min	174	220	232	222	206	204	285	288
Max	224	302	283	282	239	237	383	380
Mean	196.8	266.8	251.6	247.4	228.6	230.2	345.1	351.6
Median	200	287	250.5	245.5	231	232	351.5	356.5
Std	15.7	32.6	14.4	14.8	7.9	7.5	29.3	24.9
**Cardinality of** $k_{\mathrm{vivo}}^{\max}$								
	262	371	322		265		427	

*Note*: The statistics are determined over the different experimental approaches, namely, growth conditions and knock-out (KO) strains that have undergone adaptive laboratory evolution (ALE). In the case of KO stains ALE, the results from two replicates are specified as approach (1) and approach (2).

**Fig. 3.**
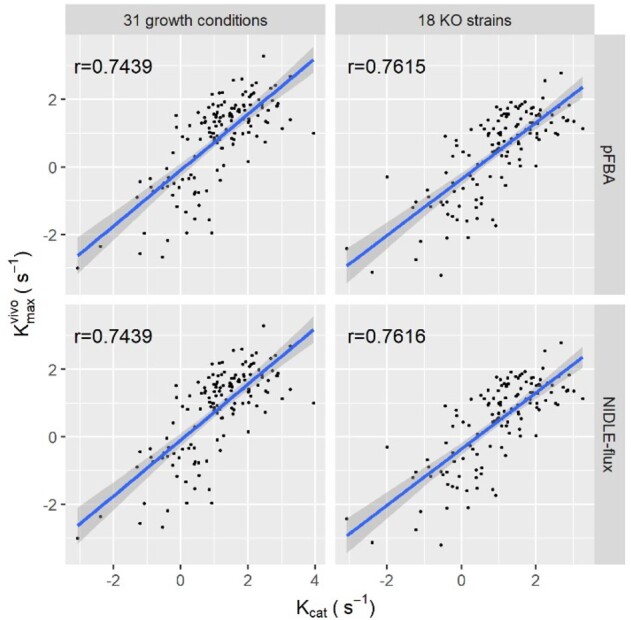
Concordance between estimates of $k_{\mathrm{vivo}}^{\max}$ and *in vitro* kcats. The concordance is determined by Pearson correlation coefficient of log-transformed $k_{\mathrm{vivo}}^{\max}$ and *in vitro* kcats from pFBA and NIDLE-flux with 31 growth conditions and 18 strains. All correlations are significant, with *P*-values < 10^*−*^^10^

Next, we compared the values of $k_{\mathrm{vivo}}^{\max}$ estimated by both pFBA and NIDLE-flux, with the constraints from 31 growth conditions, to available data on 139 *in vitro* $k_{\mathrm{cat}}$ values. The Pearson correlations for both comparison were 0.74 (*P*-value<$10^{-16}$,). In the case of the 18 strains, $k_{\mathrm{vivo}}^{\max}$ estimated by both pFBA and NIDLE-flux could be compared with 133 *in vitro* $k_{\mathrm{cat}}$, resulting in Pearson correlation coefficients of 0.76, respectively (both *P*-value <$10^{-16}$, see Fig. 3). Therefore, NIDLE-flux does not compromise the quality of the maximal *in vivo* enzyme catalytic rates, while it results in increasing its size due to the maximization of non-idle enzymes.

We found that, of the 169 enzymes for which NIDLE-flux, but not pFBA, resulted in estimated $k_{\mathrm{vivo}}^{\max}$ values, Tryptophanase (L-tryptophan), Valine transaminase and Glycolate transport via sodium symport exhibited largest values of 64.32 and 12.72 and 5.31 s^-^^1^. The remaining 166 enzymes showed values smaller than $1.6\times 10^{-6}\;$s^-^^1^, with a minimum of $3.2\times 10^{-9}$ s^-^^1^ for Enoyl-[acyl-carrier-protein] reductase (NADH) (n-C12:0). In comparison, for the $k_{\mathrm{vivo}}^{\max}$ estimated by both pFBA and NIDLE, the minimum value was $6\times 10^{-8}$ s^-^^1^ for l-threonine dehydrogenase (see Supplementary Fig. S6).

### 3.4 Validation of the estimates by integration of metabolite concentrations

Based to the available data on metabolite concentrations, we next used the estimates of $k_{\mathrm{vivo}}^{\max}$ to determine selected reaction fluxes in different growth conditions and verified their concordance to flux predictions from the two approaches, pFBA and NIDLE-flux. This verification served as an additional control for the meaningfulness of the estimated fluxes by NIDLE-flux. The flux carried by a reaction in each condition was assumed to follow Michaelis–Menten-like kinetics:

(30) vi∼=kvivo,imax·E· ∏jsjKjmj1+∏jsjKjmj

where E denotes the enzyme concentration, $K_{j}$ represents Michaelis–Menten constant, $s_{j}$ is the concentration of the jth substrate and $m_{j}$ is the respective stoichiometry of the substrate. For the three different uptake conditions, following this approach, fluxes for 260 and 370 reactions (cardinality of $k_{\mathrm{vivo}}^{\max}$) were analyzed in pFBA and NIDLE-flux, respectively. For the scenario of growth on glucose, as a major carbon source, the median Pearson correlation between the fluxes ${\tilde{v}}_{l}$, estimated based on kinetics and the fluxes v, obtained from the constraint-based approaches, was 0.97 (*P*-value = $5.21\times 10^{-10}$) for both pFBA and NIDLE-flux. In the case where the sole carbon source was glycerol, the correlation was 0.37 (*P*-value = 6.4$\times \;10^{-6}$), while with acetate as a sole carbon source, the correlation was 0.35 (*P*-value = 3.9$\times \;10^{-5}$), for both pFBA and NIDLE-flux. In all three cases, fluxes estimated from NIDLE-flux and pFBA were equally concordant to fluxes calculated from the assumed kinetics and concentration data, obtained from matching experiments. This finding provided further support for the principles used in the design of NIDLE-flux to estimate fluxes.

### 3.5 Precision of the estimates of the maximal *in vivo* enzyme catalytic rates

Next, we examined the precision of the flux estimates obtained from NIDLE-flux. To this end, we inspected the variability of flux predictions at the optimal number of active reactions that carry the smallest total flux. pFBA is known to result in very small variability, leading to high precision in the estimated $k_{\mathrm{vivo}}^{\max}$. For 31 different growth conditions, FVA was performed with NIDLE-flux by constraining the total sum of integer variable and the total sum of fluxes to the respective optima (see Section 2). For an average of 85% of reactions, the flux variability was less than 1% of their net flux, and for 84% of reactions, the flux variability was less than 0.01% (Supplementary Fig. S7), indicating the robustness of the estimates of fluxes as well as the associated $k_{\mathrm{vivo}}^{\max}$.

### 3.6 Effects of penalizing reactions with inactive GPR rules

We also considered another version of NIDLE-flux, termed NIDLE-penalized, in which the difference between the number of reactions that carry flux and are associated with active GPR rules and those that carry flux but are associated with inactive GPR rules is optimized. This scenario aims to avoid potential bias due to activation of reactions for which there is no proteomics evidence to carry flux (as a contrasting case of the so-called idle enzymes); note, however, that we already showed that these reactions carry three orders of magnitude smaller flux in comparison to those that carry flux and are associated with active GPR rules (see Section 3.1). Our findings with the data from the 31 growth conditions, indicated a further slight increase in the number of $k_{\mathrm{app}}$ values that can be estimated, ranging from 219 to 309, allowing altogether the estimate of 382 unique $k_{\mathrm{vivo}}^{\max}$ values (Supplementary Fig. S2 and Supplementary Table S5). Thus, NIDLE-penalized resulted in an increase of additional 21 $k_{\mathrm{vivo}}^{\max}$ values over the proposed NIDLE-flux approach. Analogous observations hold for the case with 18 strains, where the number of the estimated $k_{\mathrm{vivo}}^{\max}$ is 466, i.e. by 50 larger than the number obtained from NIDLE-flux (Supplementary Fig. S2 and Supplementary Table S5). The correlation of the estimated $k_{\mathrm{vivo}}^{\max}$ for the 31 growth conditions and 18 strains with available data on *in vitro* $k_{\mathrm{cat}}$ values resulted in Pearson correlation values of 0.69 and 0.73 (*P*-values < $10^{-15}$), respectively (Supplementary Fig. S3). Therefore, NIDLE-penalized provided a further slight increase over the size of the estimated kcatome without compromising the correlations to data from public databases.

## 4 Discussion

The constraint-based framework has been shown useful not only for understanding the genotype-to-phenotype mapping, but also for estimating key characteristics of enzymes, including their turnover rates and regulation by using advances from high-throughput molecular profiling technologies (Davidi *et al.*, 2016; Hackett *et al.*, 2016; Heckmann *et al.*, 2020). Key to the application of the recent constraint-based approaches to estimate proxies for the kcatome of an organism is having access to flux estimates which are in line with physiological constraints.

Here, we were motivated by the observation that cells tend to optimize the usage of expensive resources, like enzymes, and posed the problem of flux estimation as an optimization problem in which we maximize the number of reactions that carry flux and are associated with expressed enzymes. This problem is tantamount to optimizing the number of non-idle enzymes. In doing so, we did not impose thresholds on protein abundance above which an enzyme is considered active; moreover, we also did not assume proportionality of flux and enzyme abundance (Robaina-Estévez and Nikoloski, 2015), usually followed in the field of context-specific metabolic modeling (Robaina-Estévez and Nikoloski, 2014). As a result, we did not bias the results with respect to the measured abundance of enzymes. Nevertheless, NIDLE-flux can readily be extended to a weighted version, if such assumptions are more suited in other applications. We also considered a variant of NIDLE-flux, which we refer as NIDLE-penalized, that penalizes the number of reactions with inactive GPR rules that carry flux.

Through a series of comparative analyses, we showed that the fluxes estimated by NIDLE-flux were in qualitative agreement when considering the reactions deemed active by pFBA and ^13^C-MFA in two recently considered scenarios, from different growth conditions as well as different knock-out strains that have undergone adaptive laboratory evolution. Due to its formulation, NIDLE-flux activates more reactions with measured abundance of the associated enzymes, thus allowing an increase in the size of the estimated kcatome for a studied organism. Moreover, we showed that the results remain robust and allowed for a further, slight increase in the size of the estimated kcatome. However, NIDLE-flux is based on a MILP formulation that is more computationally expensive in comparison to the commonly applied pFBA; yet, the available solvers can handle the size of genome-scale computational models that facilitate the integration of large-scale proteomics data.

Our results demonstrated that NIDLE-flux, in comparison to the contenders, leads to an increase of the estimated proxy for the kcatome of *E.coli* by at least 1.4-fold, without reducing the agreement to *in vitro* kcat values. Moreover, in comparison to the contending approaches, the fluxes estimated by NIDLE-flux were in better agreement with fluxes determined from the estimated maximal *in vivo* enzyme catalytic rates, proteomics data and metabolite concentrations measured in the same experiments (while assuming a plausible Michaelis–Menten-like kinetics). Finally, extensive analysis of robustness, due to the thresholds used for considering a reaction active, and analysis of precision, due to the existence of alternative steady-state flux distributions compatible, we showed that the estimated values for the maximal *in vivo* enzyme catalytic rates show small variation. Therefore, flux estimated from NIDLE-flux can be used to catalogue proxies for the kcatomes of different species (and their natural variability) and employ them in biotechnological applications and future endeavors of parameterizing large-scale kinetic models of metabolism.

## Funding

This work was supported by the MELICOMO project [031B0358B] of the German Federal Ministry of Science and Education to Zoran Nikoloski. This project has received funding from the European Union's Horizon 2020 research and innovation programme [862201 to Zoran Nikoloski].

*Conflict of Interest*: The Authors declare that there is no conflict of interest.

## Data availability

The data underlying this article are publicly available and their corresponding references provided withing the article.

## Supplementary Material

btab575_Supplementary_Data
