# Supplementary material for: Maximization of non-idle enzymes improves the coverage of the estimated maximal in vivo enzyme catalytic rates in Escherichia coli
Source: Bioinformatics. 2021 Aug 6;37(21):3848–55. doi: 10.1093/bioinformatics/btab575 (PMC10186155; doi:10.1093/bioinformatics/btab575)
Supplement: btab575_Supplementary_Data [file btab575_supplementary_data.zip › Supplementary information.pdf]

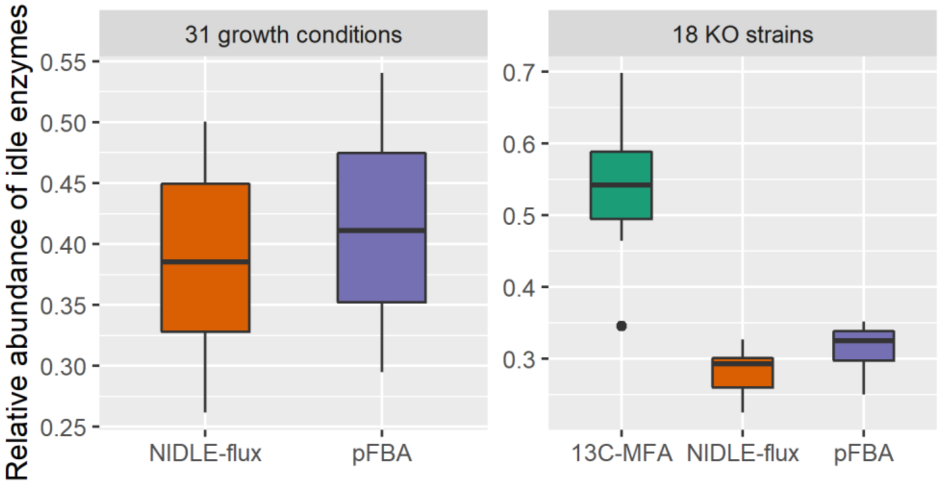

Supplementary Figure 1. Relative abundance of idle enzymes between approaches. Relative abundance of idle enzymes is the ratio of abundance of enzymes predicted to be idle to the total abundance of expressed enzymes. Comparison of relative abundance of idle enzymes in (A) 31 growth conditions, with no significant difference (p-value=0.14), and (B) 18 KO strains, there is a significant difference between NIDLE-flux and pFBA and also between NIDLE-flux and ^{13}C-MFA (p-value=0.002 and $1.5 \cdot 10^{-9}$, respectively).

Robustness Analysis

The threshold of flux value to be considered as active is a parameter of NIDLE-flux, and was set to 10^{-10} mmol/(gDWh)$^{-1}$ because this value was used in ^{13}C-MFA study (Heckmann et al., 2020). To see how NIDLE-flux behaves with larger threshold, we used the minimum threshold with which NIDLE_penalized could be implemented. To this end, we examined how the change of the threshold value (to 10^{-5} mmol/(gDWh)$^{-1}$) for considering the activity of a reaction affects the $k_{app}(C)$ and k_{vivo}^{max} estimation.

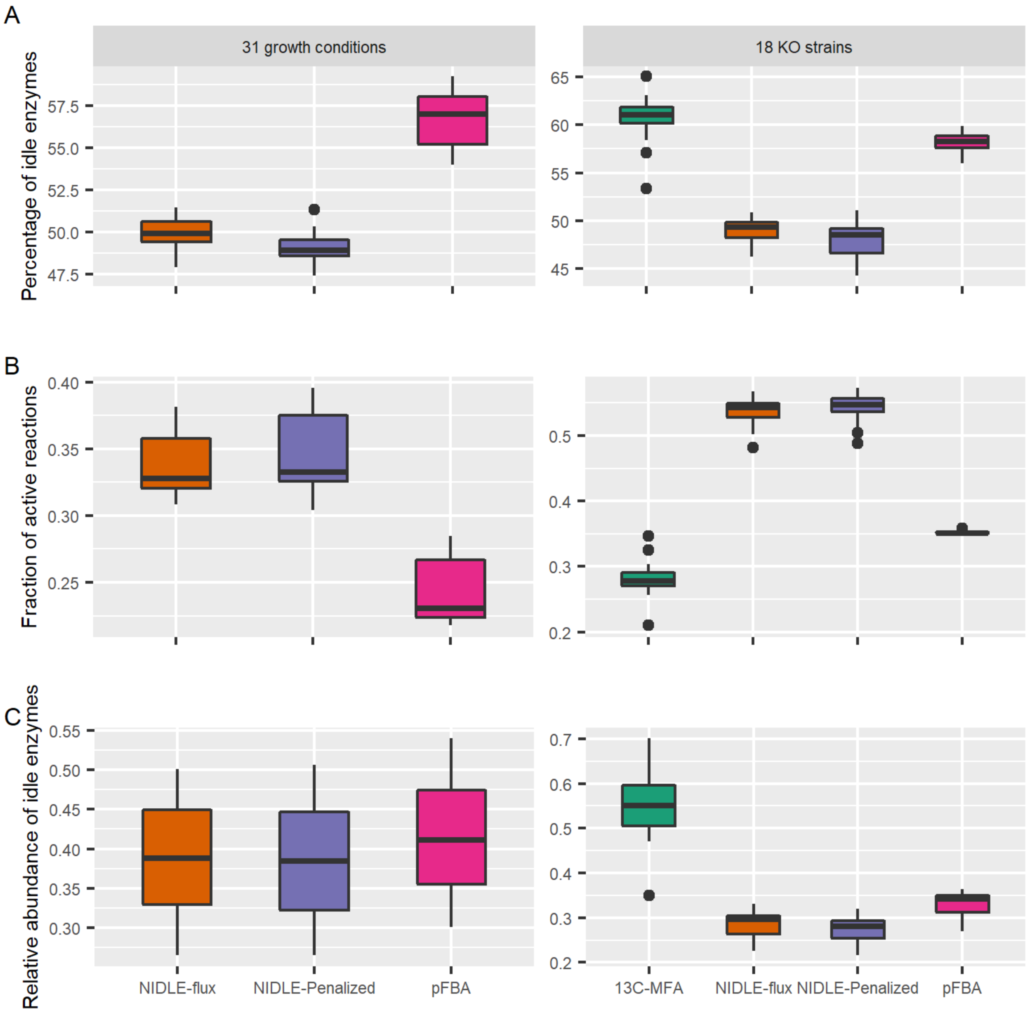

Supplementary Figure 2. Comparison of contending approaches based on idle enzymes by using threshold =10^{-5} mmol/gDW/h. NIDLE-flux and NIDLE_penalized were compared to pFBA on data from 31 growth conditions, and in addition with ^{13}C-MFA on data from 18 strains with respect to percentage of idle enzymes (A), showing a significance difference to other two approaches (p-value<10^{-9}). With the highest ratio of number of reactions carrying non-zero flux to the number of reactions associated with expressed enzymes (B), NIDLE-penalized flux shows its ability to activate more reactions per enzyme with measured abundance in comparison to the two contending approaches(p-value<10^{-10}). In the case that the 31 conditions data are used, here is no significant difference between relative abundance of idle enzymes (C) estimated by pFBA and NIDLE-flux (p-value=0.13, and 0.12 for its variant). However, on data from 18 strains, NIDLE-flux and the other version are significantly different to other two approaches with respect to the relative abundance of idle enzymes (p-value=0.0003 and 2 $\cdot 10^{-5}$).

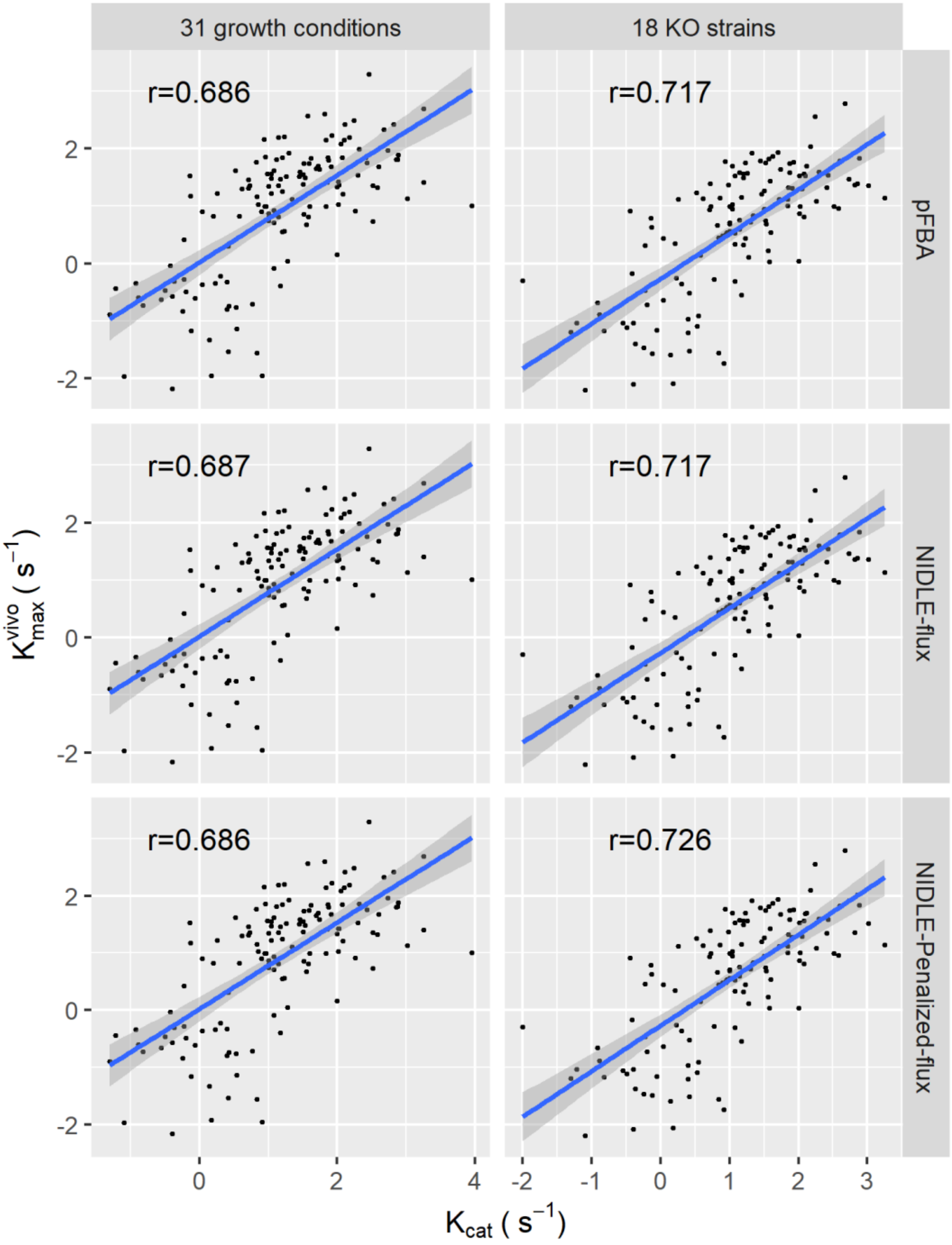

Supplementary Figure 3. Pearson correlation between estimates of k_{vivo}^{max} and *in vitro* kcat. The analysis is performed by using threshold=10^{-5} mmol/gDW/h for a reaction to carry flux, and by comparing the two different versions of NIDLE with pFBA approach. In all tested cases, the p-value is smaller than 10^{-15}, showing the good concordance between the estimates from different implementations and the in vitro kcat.

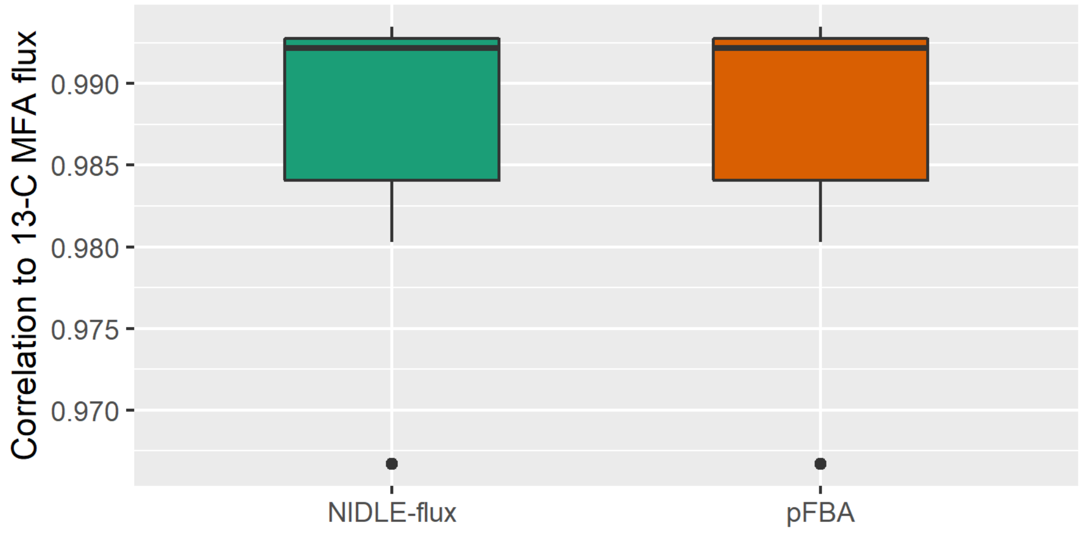

Supplementary Figure 4. Correlation between fluxes estimated by pFBA and NIDLE to the fluxes estimated by ^{13}C-MFA under 18 strains. The reactions considered are those deemed active (above the threshold 10^{-10} mmol/(gDWh)$^{-1}$) by all three approaches.

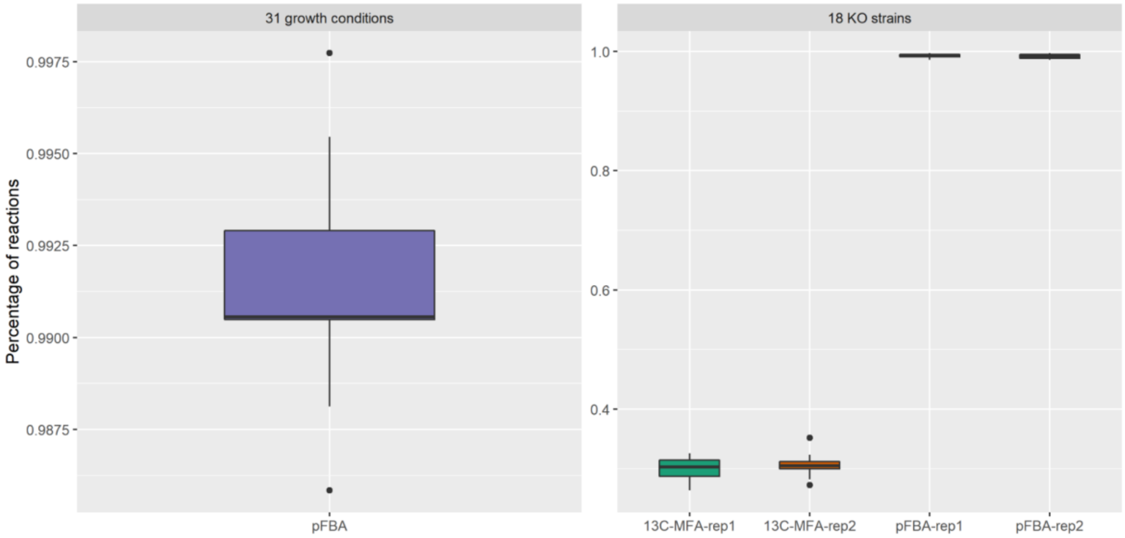

Supplementary Figure 5. Overlap of active reactions in contending approaches and NIDLE-flux. Shown are the percentage of the active reactions from NIDLE-flux that are also active in each of the two contending approaches. More than 99% of the active reactions in pFBA were also active in NIDLE-flux. Only 30% of the reactions deemed active in 13C-MFA were also active in NIDLE-flux.

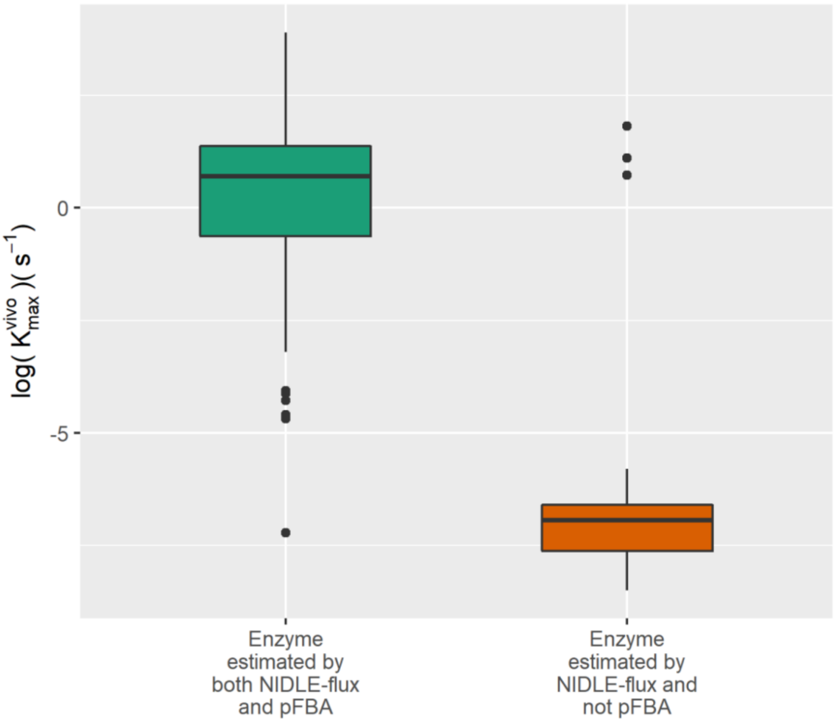

Supplementary Figure 6. k_{max}^{vivo} values estimated by NIDLE-flux and pFBA. The boxplots shown above summarize the values of $log(K_{max}^{vivo})$, that were estimated by NIDLE-flux and pFBA, where the left boxplot is for 167 enzymes estimated by both approaches, and the right one for 260 enzymes estimated by NIDLE-flux and not pFBA.

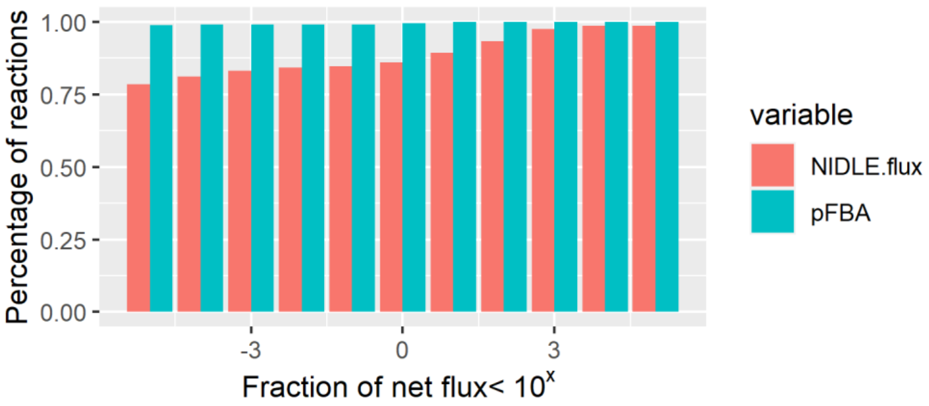

Supplementary Figure 7. Comparative analysis of cumulative distribution of flux variability. Shown are the cumulative distributions of flux variability from pFBA and NIDLE-flux. The x-axis denotes flux variability, determined as the ratio between the flux ranges, determined by FVA variants specifically designed for pFBA and NIDLE-flux, and the flux used in the calculation of k_{max}^{vivo}.

Reference

Davidi, D., Noor, E., Liebermeister, W., Bar-Even, A., Flamholz, A., Tummler, K., Barenholz, U., Goldenfeld, M., Shlomi, T., & Milo, R. (2016). Global characterization of in vivo enzyme catalytic rates and their correspondence to in vitro kcat measurements. *Proceedings of the National Academy of Sciences of the United States of America*, *113*(12), 3401–3406.

Heckmann, D., Campeau, A., Lloyd, C. J., Phaneuf, P. V., Hefner, Y., Carrillo-Terrazas, M., Feist, A. M., Gonzalez, D. J., & Palsson, B. O. (2020). Kinetic profiling of metabolic specialists demonstrates stability and consistency of in vivo enzyme turnover numbers. *Proceedings of the National Academy of Sciences of the United States of America*, *117*(37), 23182–23190.
